# Medical News and Miscellany

**Published:** 1899-11

**Authors:** 


### Medical News and Miscellany.

---

**The Injunction Suit,** Louisiana vs. Texas Quarantine Authorities, has been postponed. It will be tried in December. Dr. Blunt, State Quarantine Officer, expresses himself as confident of beating it.

---

**The New Orleans Polyclinic,** thirteenth annual session, opens November 20, 1899; closes May 10, 1900. Every inducement in clinical facilities for those attending. The specialties are fully taught. Further information, New Orleans Polyclinic, New Orleans, La.

**The writer** wishes to say that practitioners will find the preparations of the Wayne Elixir Company reliable and useful. Try them.

---

**Courier of Medicine Visiting List** for 1900. Physicians will find this list handy and to meet requirements. Price, post paid, 75c. Courier of Medicine, St. Louis.

---

**Warner's Pocket Medical Dictionary,** just the thing for students. We have received a copy of this handy little volume—a more extended notice of which was published in our August number, and can recommend it. Price $1 net.

---

**Erratum.**—In the October number of the JOURNAL the second and third lines in the editorial announcement of Dr. Daniel's book got transposed in making up the form, after proof had been revised, thereby getting the meaning a little mixed.

---

**Dr. Daniel's Book:** "Recollections of a Rebel Surgeon," etc., it is expected will be ready to deliver before our next issue. It has been delayed, unexpectedly, by the non-arrival promptly, of the paper which was made to order for it. Send in your orders, Doctors, if you would secure a copy—$1.10, post or express paid.

---

**Anti-Phymin** ("against tubercle.") We publish herewith full page advertisement of the Anti-Phymin Co., of Austin, sole sales agents for Dr. L. W. Cock's "rational remedy for tuberculosis." The formula would indicate that the remedy will meet the indications—and experience, so we are assured—has abundantly demonstrated it.

---

**A bill,** accompanied with a personal letter, was sent October 10 to every one of our delinquent subscribers. This involves an immense amount of labor and expense, and cannot be done again soon. While very many promptly responded, there are very many who have not responded at all. We hope those who have not remitted will kindly help us out.

---

**H. Stephen Smith, M. D., D. D. S.,** has located at Austin and opened a dental office at 612 Congress Avenue. Dr. Smith is the son of our old residenter, the well known Dr. Q. C. Smith, and was raised in Austin. He is an M. D. from Jefferson Medical

College (class 1898-9) and a D. D. S. from Chicago Dental College, and is well equipped for the practice of Dentistry.

---

**Change of Address.**—Dr. R. H. Anderson has removed from Lorena, to Waco, Texas; Dr. J. A. Watson from Bartlett, Texas, to Shawnee, I. T.; Dr. J. C. Burns from Gainesville, Texas, to Cliff, I. T.; Dr. A. J. James from Cleburne to Houston; Dr. G. W. Parsons from Foster to Conroe, Texas; Dr. T. E. Standifer from Eolian, Texas, to Cheyenne, O. T.; Dr. H. S. Wilder from Weatherford, Texas, to Francis, O. T.

---

**For Sale.**—$1600 practice in Central Texas. Black land, thickly settled; nice village; no opposition. $500 takes nice house, lot, barn, office, drugs and this practice. Aim to retire from general practice. This is a bargain. If you are business, write. Terms, $200 cash, balance to suit. Address,

DR. ——— B.,
Care of TEXAS MEDICAL JOURNAL.

---

**The University Regents.**—The election of Regent W. L. Prather, of Waco, to the Presidency of the University of Texas creates a vacancy on the Board of Regents. A decent regard for the claims of the medical profession and for the proprieties,—the University having a flourishing medical department, would suggest to the Governor that the vacancy be filled by the appointment of a *physician*,—a member of the State Medical Association; there is no representative of the medical profession on the board.

---

**The Antikamnia Chemical Co.** are putting on the market two new tablets: one of laxatives and alteratives with Antikamnia and one of ditto, ditto and quinine. A glance at the formulæ, which are published on each package, will show the wide range of their therapeutic application. If by any chance the reader has not been "sampled" a card to the Antikamnia Chemical Co., St. Louis, will "fetch" both sample and literature on subject. Attention is called to their full page advertisement in this issue—front form.

---

**Transactions Texas State Medical Association.**—Thirty-first annual session, held at San Antonio, April 25-28, 1899. Dr. J. T. Wilson, President; Dr. H. A. West, Secretary. We have received the Transactions. It is a neat volume of 350 pages, cloth and gilt, containing the minutes of the meeting—all the papers which were read, all the addresses delivered, etc. It is up to its usual standard of excellence, and was gotten out by Von Boeckmann, Schutze & Company at Austin—the leading establishment of Texas. The Von Boeckmann Company have secured the contract on competitive estimates for many years.

---

**The Medical Practice Law in Texas.**—The JOURNAL receives many inquiries on the subject of the medical practice law, there being a great diversity of opinion among lawyers as to its meaning: it is ambiguous and conflicting. In our January, 1896, number (Vol. XI, pages 382 to 390), we gave the law in full, both the civil and criminal statute, and published the decision in the Kennedy case. Kennedy had not registered his diploma; nor had he a certificate from any board. We will say here, for the information of those interested, that the law does *not* require any person to be examined by any board and obtain a certificate who has a diploma from "an accredited medical college, chartered in the State in which it is located"—provided the diploma is registered in the court records in the district in which the holder is practicing. Such person is a legally qualified physician, and can recover his fees by law. There is no decision on this point to my knowledge. I don't know that it has ever been controverted. The law clearly makes a certificate the *alternative* of a registered diploma.

---

### National Educational Association.

---

ANNOUNCEMENT—AMENDED SPELLING.

SECRETARY'S OFFICE, WINONA, MINN., Aug. 1, 1898.

The Department of Superintendence of the N. E. A., at its meeting in Indianapolis, Ind., Feb. 17, 1898, appointed a committee consisting of Dr. Wm. T. Harris. United States Commissioner of Education, Washington, D. C.; Dr. F. Louis Soldan, Superintendent of Schools, Saint Louis, Mo., and T. M. Balliet, Superintendent of Schools, Springfield, Mass., to recommend a list of words with simplified spelling for use in the published proceedings of the Department.

The report of the committee was duly made and the spelling so authorized was used in the published proceedings of the meeting of the Department held in Chattanooga, Tenn., Feb. 22-24, 1898.

At a meeting of the Board of Directors of the N. E. A. held in Washington, D. C., July 7, 1898, the action of the Department of Superintendence was approved and the list of words with simplified spelling adopted for use in all publications of the N. E. A. as follows:

Program (programme); tho (though); altho (although); thoro (thorough); thorofare (thoroughfare); thru (through); thruout (throughout); catalog (catalogue); prolog (prologue); decalog (decalogue); demagog (demagogue); pedagog (pedagogue).

You are invited to extend notice of this action and to join in securing the general adoption of the suggested amendments.

IRWIN SHEPARD, Secretary.

---

### The Gospel of Work.

Dr. I. N. Love, the editor and owner of Love's *Medical Mirror,* believes in the gospel of work, and constantly endeavors to evidence the fact in his columns. We present herewith, with a view to its emphasis, an editorial from the May, 1899, issue, page 252, of the *Mirror*: "Congenial work is surely next to an unselfish love the greatest pleasure in life, and properly pursued conducive to longevity. Every worker, to be a success must love his work, and love it more for its own sake than for any reward in the way of wealth or fame which follows. This real love of the work over and above the recompense, makes the true artist, whether he wield the brush, the pen, the scalpel or the plow. Rudyard Kipling, the world's second Shakespeare and first literary artist of the day (thank God, his life has been spared us), in his little poem entitled 'L'Envoi,' presents us the heaven of the honest workman as epitomized by the painter, as follows:

"When earth's last picture is painted and the tubes are twisted and dried,
When the oldest colors have faded, and the youngest critic has died,
We shall rest, and, faith we shall need it—lie down for an aeon or two,
Till the Master of All Good Workmen shall put us to work anew.

And those who were good shall be happy; they shall sit in a golden chair;
They shall splash at ten-league canvas with brushes of comet's hair;
They shall find real saints to draw from—Magdalene, Peter and Paul;
They shall work for an age at a sitting, and never be tired at all!

And only the Master shall praise us, and only the Master shall blame;
And no one shall work for money, and no one shall work for fame,
But each for the joy of working, and each in his separate star,
Shall draw the Thing as he sees it for the God of Things as they are!"

"Strenuous endeavor in building up a medical journal brings definite results, and this explains the phenomenal success of Love's *Medical Mirror.* If you want a monthly reflector of the medical profession and its progress, which is luminous, live, not perfect, but up-to-date and adapted to doctors on earth today, send for sample copy, or if you are willing to take it on faith, send one dollar to 'Love's Mirror Co.,' 4661 Maryland Avenue, St. Louis, Mo."

### Selected Formulae.

**Bites of Insects, etc.**—

℞ Liq. ammon.,
Collodii.................................aa gtt. 90
Acid salicyl .......................... gr. 3

M. Sig. Apply a drop upon each bite.

℞ Naphthaline,
Vaselin, aa q. s. ad saturated.

M. Sig. Rub in a few drops every three or four hours. (For bites of insects and bee stings.)—*Louisville Medical Journal.*

**Tuberculous Laryngitis.**—To relieve the vomiting following as the result of a morning's bout of coughing:

℞ Menthol,

Sulphuric ether,

Ol. pini sylvestris,

Tinct. iodii.................................aa ʒ ij

Tinct. benzoin comp......................ad ℥ ij

M. sig. Ten or more drops to be dropped on the sponge of an oronasal inhaler, to be worn indoors as often and as long as is convenient.—*Fowler*, *Inter-Colonial Medical Journal of Australasia.*

**Asthma.**—

℞ Ext. euphorbiæ piluliferæ.........fl., fl. ℥ j

Sig. Thirty to sixty drops as required.—*Payne.*

℞ Pulv. stramonii fol.,

Pulv. belladonnæ fol ................. aa ℥ j

Pulv. potass. nit..................... ʒ iss

Pulv. opii ............................ gr. xv

M. Sig. Burn a little and inhale the fumes.

℞ Potass. iodid.......................... ʒ viiss

Tinct. lobeliæ.........................fl. ʒ viiss

Aq. dest..................................fl. ℥ xvss

M. Sig. From a tea to a tablespoonful in a glass of beer before meals.—*Dujardin-Beaumetz Dominion Medical Monthly.*

**Tonsilitis.**—

℞ Sodium benzoate........................ ʒ 1–4

Elix. calisaya..............................aa ℥ 1

M. Sig. Teaspoonful every hour or two.—*Stevens, N. Y. Polyclinic.*

---

**Bed Sores.**—

℞ Argenti nitratis.......................... gr. 40
Aq. dest.................................. fl. ℥ 2

M. Sig. Paint red and tender spot daily. *Indications:* To be employed over bed sore spots which threaten to break. Also employed in surface ulcerates.

℞ Hydrarg. perchlor...................... gr. ij
Spir. rect................................. fl. ℥ j

M. Sig. Use locally.—*Erichsen.*

℞ Alumin,
Sodii chloridi............................ aa ℥ ss
Aq.,
Alcoholis................................. aa Oj

M. Sig. For local use, twice daily. (To prevent bed sores.) —*Forbes, Dominion Medical Monthly.*

---

**Compound Iodoform Powder for the Dressing of Uterine Ulcers.**—The *Gazette hebdomadaire de medecine et de chirurgie* gives the following:

℞ Finely sifted iodoform,
Powd. cinchona,
Powd. benzoine,
Powd. carbonate of magnesium saturated
with ess. of ecalyptus, aa equal parts.

M.—*N. Y. Medical Journal.*

---

**Palatable Effervescent Quinine.**—The *Internat. Med. Mag.* gives the following useful formula:

℞ Quininæ sulph...................... 4
Acidi citrici....................... 10
Syr. simplicis,
Syr. aurant. cort.................... aa 1
Aq. dest............................q. s. ad 20

M. Sig. Add 10 or more drops to about 50 grm. of water, in which 0.3 grm. of bicarbonate of sodium has previously been dissolved, and drink while effervescing.—*Klin. therap. Woch'schrift.*

